# Children's Laughter and Emotion Sharing With Peers and Adults in Preschool

**DOI:** 10.3389/fpsyg.2019.00852

**Published:** 2019-04-24

**Authors:** Asta Cekaite, Mats Andrén

**Affiliations:** Child Studies, Department for Thematic Research, Linköping University, Linköping, Sweden

**Keywords:** social interaction, emotion socialization practices, laughter analysis, child-adult and child-child conversations, shared norms and values

## Abstract

The present study investigates how laughter features in the everyday lives of 3–5-year old children in Swedish preschools. It examines and discusses typical laughter patterns and their functions with a particular focus on children's and intergenerational (child-adult/educator) laughter in early education context. The research questions concern: who laughs with whom; how do adults respond to children's laughter, and what characterizes the social situations in which laughter is used and reciprocated. Theoretically, the study answers the call for sociocultural approaches that contextualize children's everyday social interaction, e.g., in different institutions or homes, to study the diverse conditions society forms for learning, sociality, and socialization and development of shared norms. Methodologically, the study makes use of mixed methods: it uses descriptive statistics that identify prevalent patterns in laughter practices and, on the basis of these results, examines social-interactional situations of children's laughter in detail. It was found that children's laughter tended to be directed to children and adults' laughter tended to be directed to adults. Eighty seven percent of children's laughter was directed to other children, and adults directed their laughter to other adults 2.7 times as often as to children. The qualitative interaction analysis shows that children and adults exhibited different patterns of laughter. Children primarily sought and received affiliation through laughter in the peer group, and the adults were often focused on the institutional and educational goals of the preschool. Overall, the study shows that intergenerational reciprocal laughter was a rare occurrence and suggests that laughter between generations is interesting in that it can be seen as indicative of how children and adults handle alterity in their everyday life. By deploying multiple methods, the present study points to the importance of viewing emotion and norm sharedness in social interaction not just as a matter of communicating an emotion from one person to another, but as an intricate process of inviting the others into or negotiating the common emotional and experiential ground.

## Introduction

Laughter is a mundane phenomenon and an expression of emotion that is ubiquitous in social life. Even very young children laugh, smile, and enjoy playful and humorous events (McGhee, 1989; Dunn, 2003) and there are many funny and entertaining elements and activities in children's everyday lives. It is argued that laughter is strongly social in inviting the others to attend to and share a particular emotional stance (Jefferson et al., 1987). Of course, laughter occurs for various reasons, not all of which are associated with funniness and humor. Nevertheless, laughter is, as any other expression of emotion, a significant feature of social life, and its occurrence and use by children is guided by various normative expectations and local values (Dunn, 2003; Cekaite, 2018). Studies, taking a social perspective on emotions, show that its occurrence, form and meaning are shaped deeply by the presence of others, roles, relationships, activities, and other contextual features (Glenn, 2003). Laughter is associated with social relational work, and, what we call “emotion sharing” in that it displays an emotional stance toward a particular focus of concern, and invites the interlocutor response and stance (Goodwin et al., 2012; see also Ruusuvuori, 2013).

However, little research, and especially, research that attends in detail to the social characteristics of laughter and emotion sharing, is available on children's laughter in contexts other than homes, although an increasing group of children worldwide spend a large part of their everyday life in early childhood education institutions. Such institutions are different from homes both in the activities and institutional roles involved, and they represent inherently multiparty settings, where a large number of children spend time together. It can therefore be assumed that the children's peer group constitutes a significant social and developmental arena (Blum-Kulka et al., 2004; Danby and Theobald, 2012; Cekaite et al., 2014). There is also a lack of studies on how children's use of laughter may vary depending on the type of recipient (children or adults).

The present study investigates how laughter features in the everyday lives of 3- to 5-year-old children in Swedish preschools. The overall aim is to examine and present typical laughter patterns and their functions with a particular focus on children's and intergenerational (child-adult/educator) laughter in an early education context^1^. The research questions asked are: (i) who laughs with whom—e.g., do children (and adults) laugh mainly with children or with adults? (ii) how do adults and children respond to each other's laughter? (iii) what characterizes the social situations in which laughter is used and reciprocated?

Theoretically, the study answers the call for sociocultural approaches that contextualize children's everyday social interaction, e.g., in different institutions or homes, to study the diverse conditions society forms for learning, sociality and socialization (Rogoff, 2003; Hedegaard, 2009; Demuth, 2013). Methodologically, the study makes use of mixed methods: it uses descriptive statistics that identify prevalent patterns in laughter practices and, on the basis of these results, examines social-interactional situations of children's laughter in detail. By deploying multiple methods, we will attend to psychological phenomena as complex and embedded within situated, moment-to-moment emerging embodied discursive practices of social interaction. The study aims to deepen our knowledge about emotion socialization by showing how laughter features in children's everyday life and social relations, both in children's peer group and between adult/educators and children in early childhood educational setting. It can thereby provide insights in the processes, social conditions and norms that can be influential for young children's learning to discern and express situationally appropriate emotions.

## Previous Research

### Emotional Expressions and Emotion Sharing

Emotional expressions play an important role in parent-infant interaction from the beginning of life (Trevarthen, 1985). In line with the social perspective, emotions are manifestly expressed and they are communicative phenomena (Harré and Gillet, 1994; Holodynski and Friedlmeier, 2010; Demuth, 2013). For instance, research on ontogenetic features of human development demonstrates that infants have a propensity for “emotion sharing” that involves basic practices of “expressive pointing” (Tomasello, 2019, p. 99) through which they “share information and attitudes with one another so as to build their common ground, both conceptually and emotionally” (p. 100).

Research on human social interaction has developed an empirically supported concept of emotional stance and located it within moment-to-moment development of social situation (Goodwin et al., 2012; Ruusuvuori, 2013; Goodwin, 2018). Stance-taking is conceptualized as an embodied process that involves expressions toward the specific focus of concern, and the recipient's (affiliative or disaffiliative) response to that stance. Emotional stances are configured by using multiple semiotic resources and modalities such as speech, intonation, bodily postures, and gestures (Goodwin et al., 2012). The notions of emotion stances in social interaction are closely related to emotion sharing (reciprocation) and can capture the interactive and relational work involved when people affiliate with, or avoid affiliating with each other's emotional states toward the referent of the emotional expression. In short, emotional expressions are communicative phenomena that often have both a referent (in cases of laughter, the laughable) and a recipient. For adults and children likewise, emotional stances, including laughter, are not just a matter of “expressing” an emotion but are often performed as a matter of sharing or not sharing an emotion (Bainum et al., 1984; Glenn, 2003; Cekaite, 2018).

Notably, in research on children's emotions, negative emotions have received much more attention than positive emotions. For instance, one of the important tenets of socialization and becoming a socio-emotionally competent is considered to involve mastery of emotion regulation that “has been chiefly focused on the increasing control that children exert over their frustration, anger, or distress” (Dunn, 2003, p. 337). However, whereas socialization into mastery of negative emotions tends to be seen as important because of their potential threat to the social harmony of the group, positive emotions are significant because they constitute a ground for sustained social relations and well-being. Studies suggest that children's expressions of positive emotions increase over the preschool years (Bainum et al., 1984; Barry and Kochanska, 2010). Barry and Kochanska (2010) studied children in American families longitudinally from infancy to early school age and found that expressions of positive emotions increased over time, whereas children's anger was highest at earlier ages and decreased thereafter. A possible interpretation is that a more positive and collaborative style of participation in social interaction becomes more important as children grow older and become more concerned with the establishment and negotiation of social relations such as friendship. Moreover, Sperling's (2012) study of emotion socialization in American homes with 8–12-year-old children (on the basis of video-recorded naturalistic family interactions) shows that expressions of positive emotions were three times as common as negative emotions. (cf. the frequent focus on negative emotions in research). In addition, the caregivers actively behaved in ways that prolonged the children's positive emotions by, for instance, reciprocating with their own displays of positive emotion (Bai et al., 2015), which we can interpret in terms of emotion sharing.

### Research on Children's Laughter

One major line of research on positive emotions and laughter in children, especially within developmental psychology, deals with the emergence of laughter in ontogeny, which happens around the third or fourth month of life (Ruch and Ekman, 2001). Another line of research focuses on the children's development of humor (rather than laughter as such). Children tend to laugh at humorous stimuli and produce so called “laughables,” inviting others to laugh at something that is relative to their current developmental stage (Pinderhughes and Zigler, 1985). They can play with and transform what they are learning and mastering at the time, e.g., playing with incongruent transformations of language structure, or social rules (Blum-Kulka et al., 2004; Cekaite, 2018). Joke-based humor involves more complex cognitive and pragmatic organization and children master these skills much later (McGhee, 1989). Research thus suggests that there are differences in what young children and adults consider to be entertaining and funny.

One of the prominent theories of laughter associates laughter with incongruencies. This goes back to scholars like Aristotle, Kant and Schopenhauer, and many researchers on humor agree that “humor is related to either comprehending or producing an incongruity: the simultaneous occurrence of incompatible elements or sudden contradiction of expectations” (Semrud-Clikeman and Glass, 2010, p. 1). The incongruity principle, however, does not fully explain why “some incongruities seem humorous while others do not” (Glenn, 2003, p. 21). For incongruity to be entertaining and socially appreciated, it has to be framed by communicative signals that indicate e.g., playfulness and humorous potentials (Bariaud, 1989). Yet another theory of laughter, foregrounded by Bergson (1911), argues that “laughter always implies a kind of secret freemasonry, or even complicity, with other laughers.” Laughter is thus viewed as a social phenomenon that indicates and strengthens affiliations and the development of social relations. It should be pointed out that the connection between humor and laughter is not fully straightforward, and that laughter can have various social functions (Provine, 2000): laughter can be reciprocated but it can also sometimes be treated as undesirable because it is disturbing or teasing (Andrén and Cekaite, 2016).

Research on children's laughter in social situations (rather than children's cognitive capacities in humor comprehension), and especially in preschool settings is rare, though at least in Scandinavian countries, children spend a large part of their everyday life there. One of few studies of children's laughter, conducted in a nursery school (with 3–5-year-old children) in the United States (Bainum et al., 1984) found that smiling was much more common than laughter, but that laughter became increasingly more common with age. Bainum et al. conceptualize laughter and smiling as emotionally similar, but not equal emotional expressions and show that laughing and smiling co-occurred with children's different actions and event patterns (see also Sarra and Otta, 2001; Petitjean and Gonzales-Martinez, 2015). Laughter more frequently served to emphasize the intentional activities of another child or as “a means of calling attention to certain aspects of the child's own ongoing (silliness/clowning) behavior.” (1984: 1955). Accordingly, this characterizes laughter as social, “highly sophisticated (even if unreflected) attempt to let the listener in on the ‘nonserious’ nature of the communication” (1984: 1956). Similarly, studies of children in preschool and primary school and children's prominent entertaining communicative genres based on incongruency—children's language play—show that laughter was used to invite peers' affiliation and, in such way, create exciting time-out from the institutional agenda of the educational setting (Cekaite and Aronsson, 2014; Cekaite, 2018). In all, the studies point to the importance of studying the actual social and interactional practices in which children's laughter evolves. Notably, research has not dealt much with laughter in child-adult—intergenerational—encounters.

### Social Interactional Studies of Laughter

Laughter in social interaction (between adults), its functions and organization, have been investigated in a substantial number of studies within the interaction analytic approach (Jefferson, 1979; Jefferson et al., 1987; Fatigante et al., 1998; Glenn, 2003; Glenn and Holt, 2013). Several of the findings have a significant bearing on this study. Interactional research shows that laughter is a highly ordered interactional phenomenon that has considerable variation both in its forms and functions. When someone is laughing this is heard as referring to what one is laughing about^2^ (Jefferson et al., 1987), sometimes called the “laughable” (Glenn, 2003). Henceforth in this study we will refer to laughter as being “directed at” someone. Interactional research also shows that the “laughable” can be highly varied, ranging from concrete, incongruent, actions to sophisticated jokes. Moreover, laughter is embodied, and especially gaze is important because it tends to indicate the recipient of the laughter (Markaki et al., 2010). Previous studies have described different possible responses to laughter: participants can join laughter and affiliate with it, or ignore it and offer serious responses instead (Jefferson et al., 1987). Smiling can also be used to respond to laughter. Distinctions have been made between “laughing at” (distancing at somebody/something through laughter) and “laughing with.” Affiliative effects of laughter as emotion sharing are particularly interesting in multiparty institutional settings (e.g., preschool) because in this collective organization it can contribute to local alliances and group partitions.

In the present study we combine insights from earlier research on children's laughter, and studies taking a social interactional perspective to examine children's laughter in preschool as early childhood socializing setting characterized by various participant—child-child and child-adult—constellations. We suggest that analyses of recurrent patterns, and social organization of laughter situations are relevant for our holistic understanding of the contextual embedding, normative expectations and social actors that are involved in young children's affective, and communicative socialization.

## Methods

### Setting, Data and Analytical Procedures

In Sweden, public preschools constitute a significant early education institution that has multiple goals, which include both education and care. Ninety two percent of children between 1 and 5 years attend preschool and on average, they spend 31 h a week there (Swedish National Agency for Education, 2015). The aims and work methods of preschools (the only type of early childhood education institution in Sweden) are defined by the Swedish National Curriculum that foregrounds a holistic approach to child development, learning, and emotional well-being. Preschool activities comprise play, education, and care, with a considerable emphasis on children's free play.

The data for the present study is naturalistic and was collected in two regular, public Swedish preschools for 1–5-year-old children, located in a middle-class area. The data consists of 77 h of video recordings, collected over a period of 1.5 years. The Regional Ethical Board in Linköping has approved the project^3^. 20.5 h of video-data were used for the analysis, and this was selected on the basis of containing everyday institutional practices for 3–5-year-old children. There were ~25 children and six educators in each preschool unit where data was collected and analyzed. The recordings involved a range of activities that are part of a regular day at the Swedish preschools: free play (the children are free to choose with whom and with what to play and they typically socialize in smaller groups), circle time (all or most children gather to educator-led educative activities, including snack/fruit time), book reading (one preschool educator reads a book to a smaller group of children), lunch time (smaller groups of children sit at tables in different rooms together with one or two educators), children drawing (with or without a preschool educator present), and more. During a preschool day, adults were present during educational, or practical institutional activities, such as reading, circle time, drawing, similar artwork, or mealtimes. Children's free play activities were largely conducted without close supervision by adults.

### Coding for Quantitative Analysis

Coding for the quantitative analysis was done using the ELAN freeware software. Instances of laughter were identified in the video recordings until the total exceeded 1,000 instances, yielding a total of 1,047 instances. This included any kind of laughter from adults or children, except silent laughter that has similar movement patterns but no sound is produced. It was also noted how many adults that were present when each instance of laughter occurred.

Each instance of laughter was coded to indicate (a) whether an adult or a child produced it and (b) whether the laughter was directed to an adult or a child as the recipient. This yields four categories: *child-to-child, child-to-adult, adult-to-child*, and *adult-to-adult*. In most cases the recipient of laughter corresponds to the one being looked at by the person who laughs: a child or an adult. In some cases, gaze is not directed to the recipient, but other contextual cues indicated to whom the laughter is directed. There were only a few (9) instances of laughter (all produced by children) that didn't seem to be directed to someone else, and these were excluded from further analysis (leaving 1,038 cases in total). For instances of child-to-adult laughter, we also coded whether the adult's response to this laughter was categorized as either *affirming, no response*, or *rejecting*. These categories are described further as part of the analysis.

## Quantitative Analysis

This section provides descriptive statistics of overarching patterns in the data. It serves as a background to the qualitative analysis, where findings from the quantitative part are unpacked by showing the underlying interactional dynamics that are involved in the institutional context of the preschool. In this way, the quantitative and the qualitative parts of the analysis complement each other.

One thousand and thirty eight instances of laughter were identified in the 20.5 h of data that was analyzed. There were 891 instances (86%) of child laughter and 147 instances (14%) of adult laughter. This makes child laughter six times as frequent as adult laughter, meaning that the average child will experience substantially more peer laughter than adult laughter in this preschool context.

**Figure 1:**
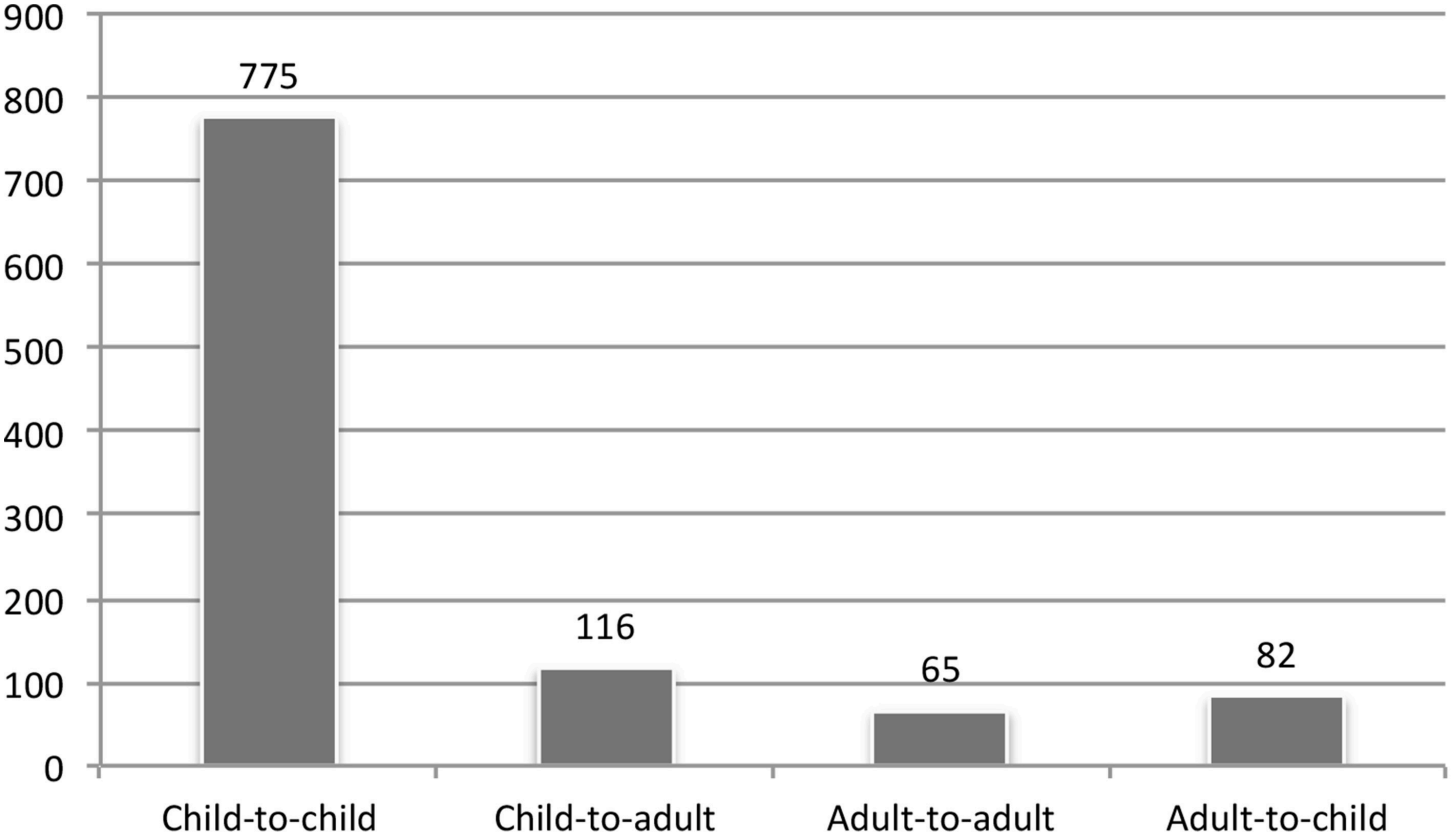
Number of instances of children's and adults' laughter to children and adults.

There is markedly more child laughter in the data, but this does not necessarily mean that each individual child laughs more often than individual adults do. It is because there are also substantially more children than adults at the preschool. At Swedish preschools there are about five children per adult (Swedish National Agency for Education, 2015). Nevertheless, this still means that each individual child will experience much more child laughter than adult laughter at the preschool. Furthermore, as shown in Figure 1, most of the child laughter was also directed to another child (*n* = 775; 87%) rather than to an adult (*n* = 116; 13%). Overall, child-to-child laughter constitutes as much as 75% of all the instances of laughter, both by adults and children, in the data. Consequently, laughter at the preschool is to a large extent a matter of peer interaction, as has also been found in a number of other areas (Cekaite and Aronsson, 2014).

**Figure 2:**
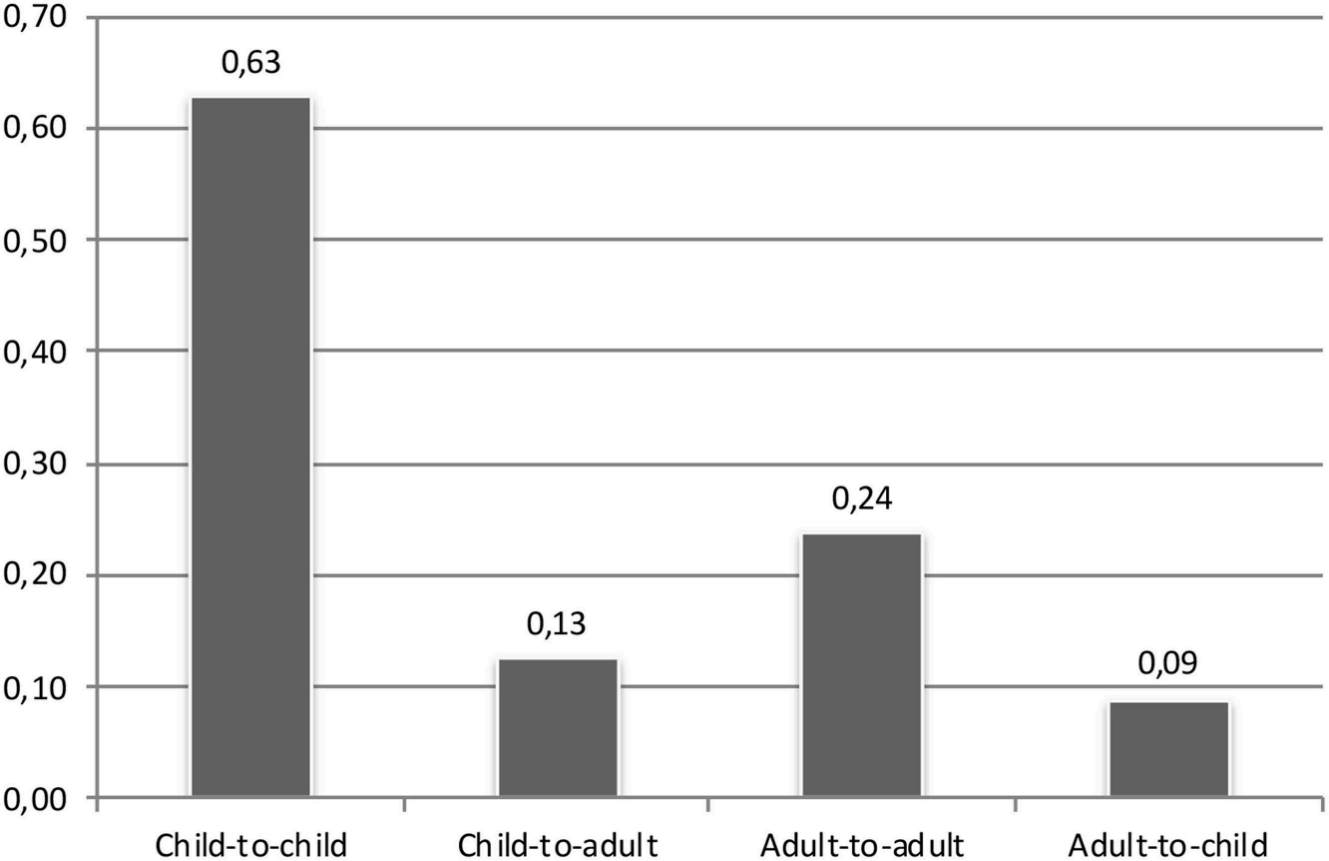
Instances per minute (when it could possibly occur) of children's and adults' laughter.

Regarding adult laughter, there were slightly more instances that were directed to a child (*n* = 82; 56%) than to another adult (*n* = 65; 44%). However, this doesn't mean that the adults at the preschool were more inclined to laugh together with children than with other adults. It is actually the other way around. To understand why one should note that two or more adults were present only for 22% of the duration of the analyzed data. This is relevant because the only time adult-to-adult laughter could possibly occur is when two or more adults are present. By contrast, child-to-child laughter could occur at virtually any time. Taking this into account, Figure 2 shows the number of instances of laughter per minute, based on the amount of time that each category could possibly occur. This reveals that adults' laughter was directed at other adults 2.7 times more often than to children, provided that other adults were around. The overall pattern that emerges is that the children tended to direct their laughter to other children and the adults tended to direct their laughter to other adults.

**Figure 3:**
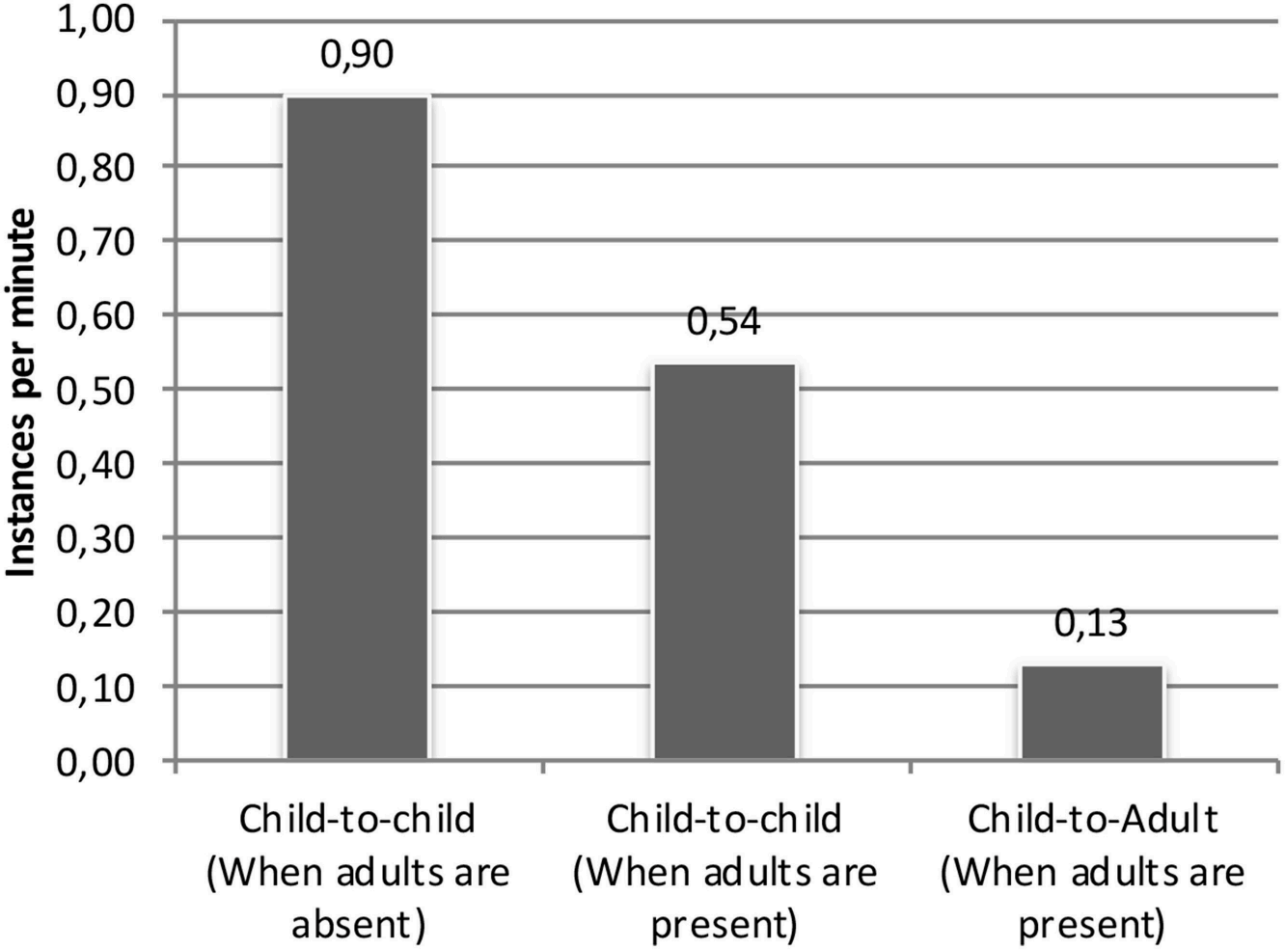
Instances per minute of child laughter when adults are present or absent.

A related finding is that children laugh less when adults are around, as shown in Figure 3. When adults are absent, child-to-child laughter occurs at a rate of 0.90 instances per minute. When adults are present, there are only 0.56 instances of child-to-child per minute (and 0.13 instances of child-to-adult laughter per minute). This finding adds another dimension to the pattern that children mainly laugh with children and that adults mainly laugh with adults. The presence of adults clearly decreases children's tendency to laugh.

**Figure 4:**
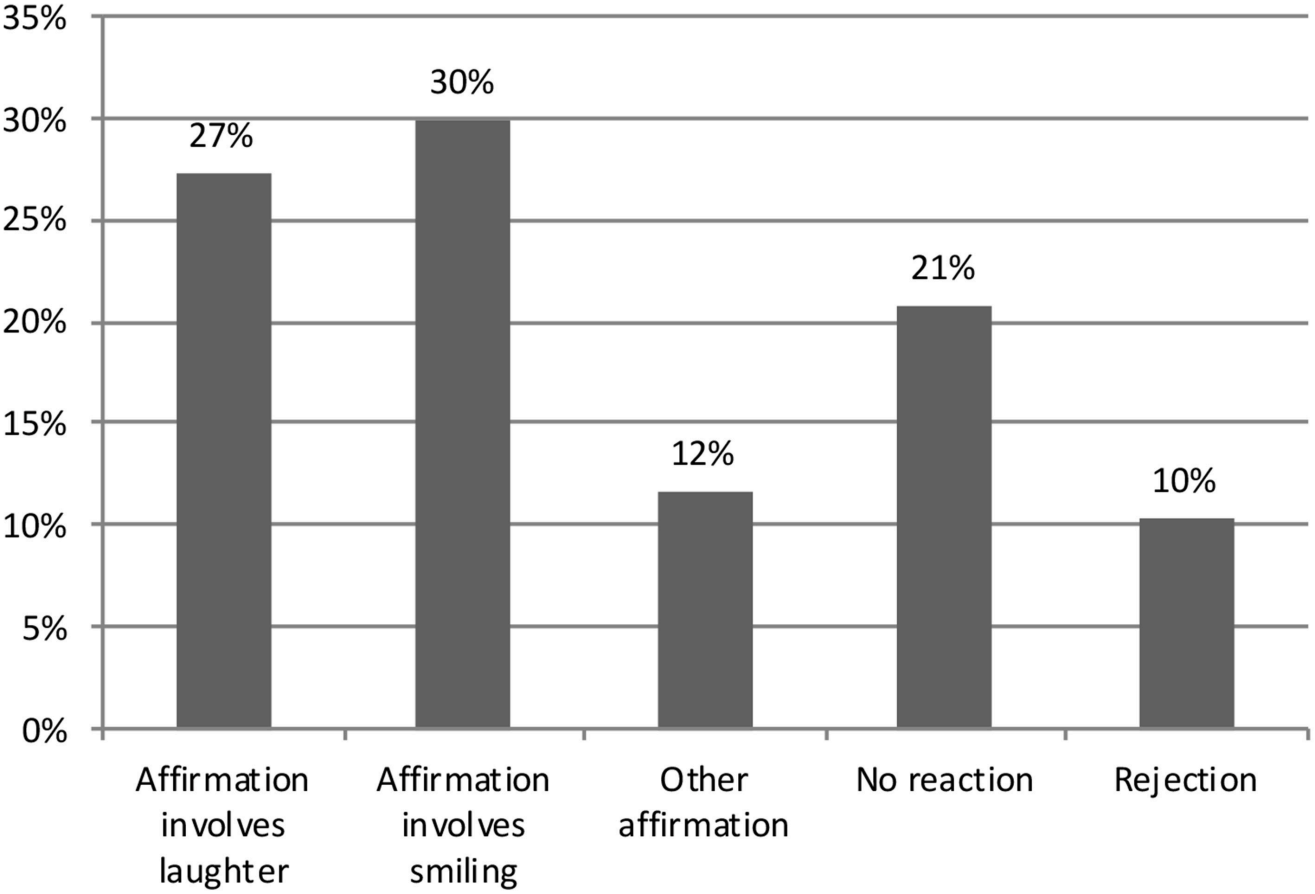
Adults' responses to child-to-adult laughter.

Why would the presence of adults have this effect? Possible reasons for this will be discussed further as part of the qualitative analysis (e.g., activity contexts where adults are present usually involved educational or other task-oriented activities, whereas children spent considerable amount of time in the peer group during free play). However, looking at the ways that adults respond to children's laughter, when the laughter is directed to adults (child-to-adult laughter), may provide some background to this. Figure 4 gives an overview of how the adults responded to child-to-adult laughter in the data. In 69% of the cases, adults responded in an affirmative way. This means that the adult reciprocated the positive emotional stance of the child's laughter in some way. Since the affirmative category was relatively large, it is broken down into three sub-categories in Figure 4. This shows that 27% of the adult responses to child-to-adult laughter were cases where the adult also laughed. In 30% of the cases, overall, the adult did not laugh, but smiled as part of their response to the child. In 12% of the cases, the response was still affirmative, but the affirmation was mainly done verbally, and did not contain laughter or smiling from the adult.

In 21% of the cases, the response from the adult was what we have coded as “no reaction.” These are cases where the actions or the speech of the adult show no manifest orientation to the child's act of laughing. For instance, an adult may be speaking and a child laughs in response to something that the adult says, but the adult keeps talking as if nothing happened.

Finally, in 10% of the cases, the response of the adult is coded as “rejecting.” This includes cases where the adult explicitly opposes or rejects the act of laughing, either because it is not deemed appropriate to laugh at a particular type of laughable (e.g., in the case of mocking) or because the very sound and engagement in laughter may disturb some ongoing activity (e.g., during activities where the children are supposed to remain silent or participate attentively in book reading).

Overall, in a majority of the cases, the child-to-adult laughter is affirmed in some way in the adults' responses. At the same time, it is relevant to note that out of *all* of the child laughter in the data, including both child-to-child and child-to-adult laughter, only 2% are cases where a child directs laughter to an adult and the adult's response also contains laughter. Shared laughter across the generations is not common^4^. This adds yet another dimension to the finding that the children mainly laugh with children, and the adults mainly laugh with adults. We will now turn to the qualitative analysis to provide some insights into the social situations that characterize children's laughter as well as adult responses to children's laughter.

## Qualitative Analysis

The focus of the qualitative analysis concerns children's laughter when it is directed to adults, peers or both. The analysis examines: (i) the activity contexts of laughter; (ii) the trajectories of entire laughter situations; (iii) responses to laughter, i.e., emotional stances, affiliation and emotion sharing. We will examine situations where adults respond to children's laughter and situations where children direct their laughter to their peers. In doing so, we aim to gain insights into the dynamics of child-to-child laughter, and child-to-adult laughter. Attention to situations where children's laughter occurs in the proximity of adults can reveal social dynamics and emotion socialization potentials linked to laughter (and emotion sharing) between various participant constellations, as well as conditions for intergenerational emotion sharing.

The qualitative analysis employs a multimodal interactional approach (Goodwin, 2000) that inductively examines how embodied social actions are accomplished in social encounters. The study analyzes what participants accomplish socially in a moment-by-moment interaction by using turn-by-turn meaning making procedures as the interaction evolves. Multimodal interaction analysis utilizes video recordings in order to examine in detail participants' verbal and embodied social actions emerging within the spatio-material configurations of the environment. Analytic orientation is on participants' verbal turn-taking and coordinated use of multiple modalities (such as gaze, touch, sound) (Goodwin, 2000). By examining how participants themselves orient to each other's actions sequentially, turn-by-turn, analysts see evidence of how the participants interpret and analyze each other's actions, and accomplish particular activities. In this study, the analytical focus was on children's and adults' laughter and what can be identified as the interactional response to laughter, displayed through the participants' publicly visible actions. To be able to exemplify the embodied features of situations of laughter, we use images, made for the specific study^5^.

### Children's Adult-Directed Laughter and Adult Responses

The children directed their laughter to adults in 13% of the cases of children's laughter, receiving various types of responses. In this section, we will look more closely at the range of adults' affirmative responses, as well as situations where there is a lack of adults' responses to children's laughter. An example that involves an adult's rejecting response is examined in a later section of the qualitative analyses, when discussing children's peer laughter (Ex. 5a–b).

### Adult Affirmation Through Smiling and Other Means of Emotion Sharing

Most often, episodes where the children's laughter was directed to adults involved situations where the adults were in charge of educational institutional activities. The child's laughter was then received in various ways, including the adult's non-response, or affirmation through smiling, or other means. Such adult responses indicate that the children's laughter and emotional stances were not rejected or disciplined by the teachers, but were corroborated by the teacher's modulated affiliative smile or by other means, or they were ignored, usually in the service of the continuous progression of the ongoing educational activity.

The children's laughter could evolve in relation to some entertaining feature of the teachers' ongoing activity, such as book-reading or story-telling, a culturally typical emotionally engaging activity (Cekaite and Björk-Willén, 2018), where the adult's actions were affectively valorized in ways that made possible or even invited the children's display of a positive stance. The children's laughter as emotion sharing was not limited to a single participant, e.g., the teacher, but could be addressed and distributed across the peer group as well. In Ex. 1, a group of 3–5-year-old children (mostly girls) sit together with the teacher in a sofa, listening to the teacher reading a story about nice monsters. Olivia laughs appreciatively toward various participants of the activity.

Ex. 1. Participants: Teacher; girls Olivia (5.1 y.);Wilma (3.2 y.).


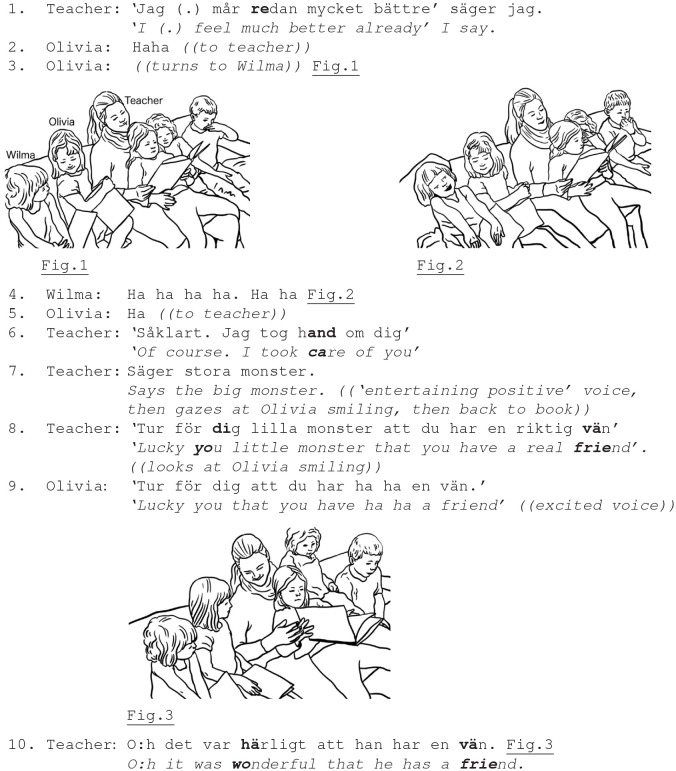


The teacher uses positive reading voice when Olivia, while looking at the book, laughs appreciatively (line 2). The teacher does not respond to the girl's laughter, but continues to read the story, and proceeds with her institutional task. Olivia then turns toward her peer, Wilma, who sits at her side, and Wilma bursts out in reciprocal laughter, affiliating with Olivia's emotional stance (line 4, Fig. 1-2). In this way, a moment of shared positive emotion is initiated and sustained by the girls. Olivia carries further her laughter and affective stance toward the teacher, laughingly turning toward her and the book (line 5). The teacher, however, continues reading; she does not reciprocate the Olivia's laughter, but she now reads with markedly positive entertaining voice, and for a short moment turns to the girl with a smile (line 7). In these embodied ways, the teacher responds to and confirms Olivia's laughter as an expression of her positive stance.

More possibilities for emotion sharing between the child and adult are established as the reading progresses. The final line of the story “*lucky you little monster that you have a real friend”* is produced by the teacher with a smile, and a gaze directed at the girl (line 8). As a result, the teacher sustains the positive stance, earlier invoked by the girl. Olivia reciprocates the teacher's positive stance by repeating the story line “*lucky you little monster”* with laughter, and the teacher once again affiliates, shares and confirms the positive emotional stance through her smile and positive voice (rather than through laughter) (line 10, Fig. 3). She also uses verbal means, an assessment “*oh it was wonderful that he has a friend”* of the story, in such way confirming emotion sharing and culturally appropriate interpretation of the story (Bruner, 1990; Cekaite and Björk-Willén, 2018).

As demonstrated, the children's laughter and various responses to it in a particular activity context support emotional attunement between the child and the adult, and in the children's peer group. The children's laughter has multiple recipients, and (in book reading context) can be directed at the adult, who may not reciprocate with laughter, but use affirmation through other means. Overly positive voice and smiles are used for both confirming and regulating the girl's emotion. In contrast, peers provide a fruitful social context for emotion sharing through reciprocal laughter. Notably, the children are socialized into, and supported in their engagement in a particular emotional interpretation of a narrative-based social relations, but with varying emotional intensity: similar emotional stances are shared with both peer and adult, but the communicative means and their affective intensity are different.

### Adults' Affirmation Through Laughter

Adults reciprocated children's laughter in, for instance, more informal situations that were not guided by educational agenda. Such laughter was, for instance, related to adults' engagement in face work when children laughed about their unexpected mistake or other type of incongruent act. In Ex. 2, during snack time, Mea is buttering her bread and is about to put a butter knife in her mouth. The teacher, who is serving the children at the same table, mildly remarks on Mea's mistake (line 1).

Ex. 2. Participants: Teacher; girls Mea (5.4 y.), Emilia (4.1y.).


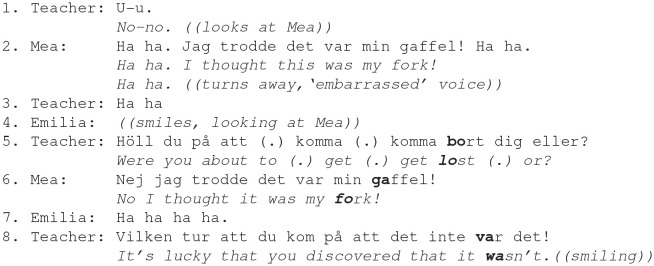


As Mea is about to put a butter knife in her mouth (by mistake), the teacher mildly and with a smile reprimands her (line 1), and the girl turns away and, in an embarrassed voice, makes an excuse, justifying her mistake “*I thought this was my fork,”* and adds some laugher (line 2). It is this embarrassed laughter that the teacher reciprocates and asks a playful question “*Were you about to (.) get (.) get lost (.) or?”* (lines 3, 5). Both the teacher and the children (Emilia and Mea) collaborate by mildly joking about and thereby justifying Mea's mistake, thus performing some face work in this slightly embarrassing situation. Shared laughter and smiles work to downgrade and mitigate the girl's inappropriate conduct (lines 2–8). The teacher, however, does not continue laughter, but makes a smiley supportive comment on Mea's account (line 8).

As demonstrated, during an extended laughter situation, various responses to the child's laugher are available and adult-affirmative responses can vary in the degree to which they affiliate the child's laughter. The child's emotional stance is temporarily reciprocated by adult's laughter used as a face-saving affiliative device. Smiling responses affirm the positive emotional stance expressed by the child's laughter and go a long way in achieving alignment between the child and the adult. However, by using a smile instead of laughter in response, the adult may display a less strong affiliation, since smiling often serves as a less strong positive expression than laughter.

### Children's Peer Directed Laughter

Children's peer-directed laughter was the most frequent category in the present data. Laughter served several functions, and its social and physical characteristics influenced how the children used it and how the preschool teachers oriented to it. Reciprocal laughter could, through emotion sharing, be used to strengthen the children's in-group alliances and emotional coalitions. Laughter in the peer group could also develop into loud laughter outbursts that disturbed and interrupted the ongoing institutional activity. The analysis shows that children laughed at something incongruent; made rudimentary jokes with various degrees of incongruity, e.g., verbal and sound play; marked their play acts and play roles, drew attention to something exciting (e.g., silliness/clowning events), or laughed appreciatively toward something in their environments (stories, talk, objects).

In this section, we will demonstrate some of the prevalent patters of children's peer laughter and describe its social interactional functions and features. In that we are interested in children's laughter and the intergenerational characteristics of children's experiences and emotion socialization in early childhood education, we here attend to children's laughter that occurs in situations when the adults are present. Thus, while the primary focus is children's peer laughter, the analysis also pays attention to the adults' conduct when children's laughter occurs in vicinity of educators.

### Child-Child Reciprocation of Laughter: Peer Affiliation and Emotion Sharing

Interaction analysis shows that during the ongoing flow of preschool activities, the children were able to discern laughable elements in their peer's talk or entertaining performances and, by using embodied resources, present them to their peers as laughables. Below we will demonstrate how children's peer group laughter is reciprocated and evolves into emotion sharing between the peer group members. The teacher, however, typically orients to the situation as a task-related one, rather than fully affiliating with the children.

In Ex. 3, three girls and a teacher are eating snacks (sandwiches, called “macka” in informal Swedish) and Mea starts talking about “Macka Packa” (a character with big ears in children's TV show). The teacher then asks Mea questions about this character and tries to initiate her explanation and narrative about it.

Ex. 3. Teacher, girls Mea (5.4 y.), Emilia (4.1 y.), Tina (4.5 y.).


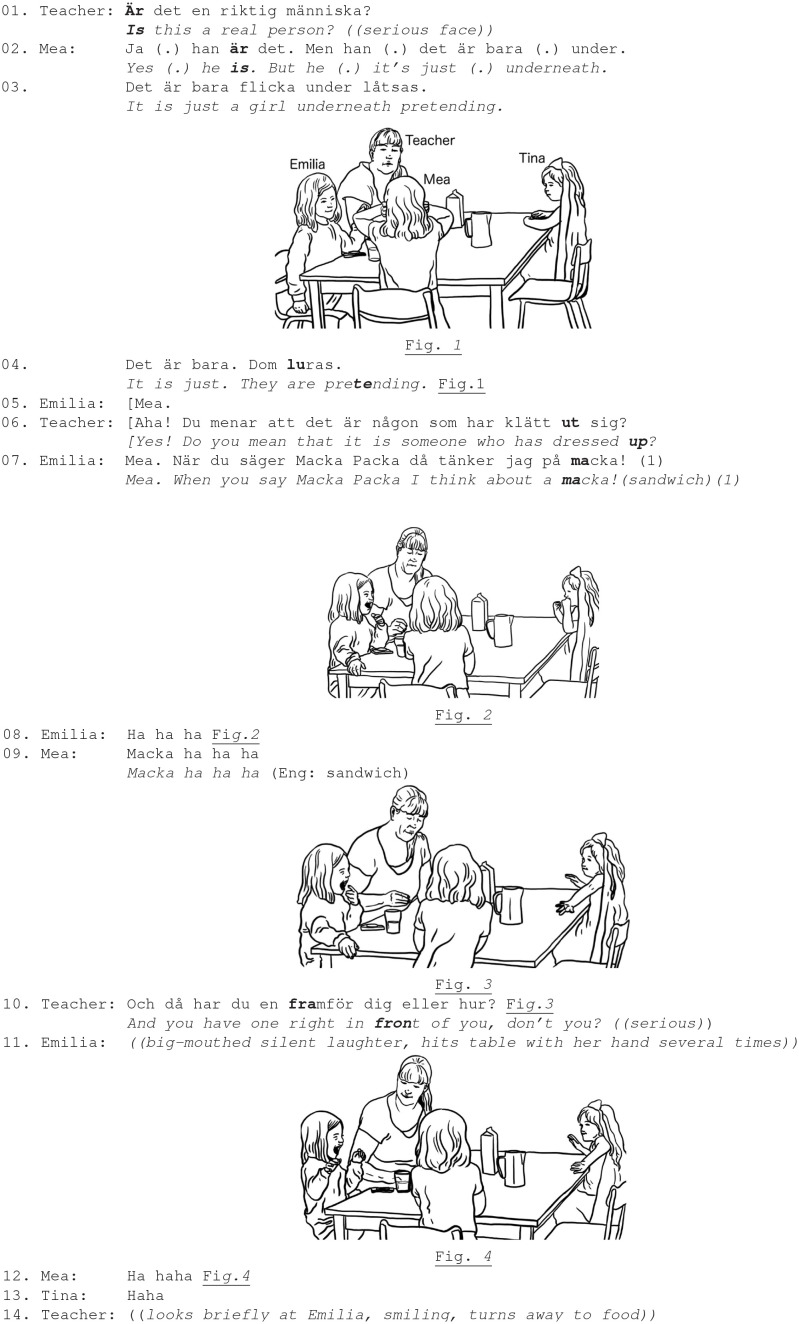


The teacher is oriented to various institutional tasks: children's eating and conversing. She sustains conversation with Mea by asking numerous questions in a serious, matter-of-fact, manner (lines 1; 6). Emilia, instead, finds some laughable potential in Mea's mentioning of “macka packa,” and identifies it as language play and pun (line 7) (Cekaite and Aronsson, 2014). While the teacher orients toward the child's factual message and creates interactional possibilities for Mea to expand her story, the peer exploits entertaining potentials of the formal aspects of Mea's talk: Emilia tells a rudimentary joke and invites the peer's laughter “*When you say Macka Packa I thinking about a macka (sandwich)”* (line 7). She creates excitement by using gaze and facial expression to invite her peer's reciprocal emotional stance (Fig. 2; 3). Emilia is shaking with laughter, moving her torso and laughing with her mouth wide open. Embodied laughter performance intensifies upon Mea joining in and reciprocating laughter. Emilia hits the table with her hand, looking at Mea and they laugh together, while looking at each other. The girls' mutual gaze attests their joint emotional stance, sustained for a rather extended time and even Tina, who was not specifically addressed by Emilia's laughter, joins their laughter (lines 8–9, 11–13; Fig. 2–4). The teacher's comment “*and you have one right in front of you”* invites a closure of the girls' laughter (line 10).

Notably, Emilia's joke was not directed at the teacher, but, since the children and the teacher together were participating in and listening to Mea's telling, one could assume that the teacher could potentially respond to the joke. The teacher, however, does not join the girls' laughter. Rather, she re-orients the girls to the institutional task of eating sandwiches (line 10). Comparing the children's and adult's responses to the joke, it is notable that the girls reciprocate laughter and build an affective alliance, sharing, and affiliating each other's emotional stance (e.g., hitting table, leaning forth, looking at each other). This social situation can be seen to strengthen social—friendship—relations between the girls. The teacher does not reject or discipline the girls' laughter, but observes the situation with a smile (line 14), aligning with the girls' experience of fun. However, she primarily deals with the progression of her institutional task (lines 10, 14).

### Children's Multiparty Laughter as Choral Emotion Sharing in the Peer Group

In a multiparty preschool context, where multiple children participated in activities together, they recurrently engaged in and invited the others (their peers and at times, teachers) to take a similar emotion stance toward some exciting object, event or act (e.g., Cekaite and Strid, in press). Such typically embodied and material artifact-linked laughter invitations to share excitement could be responded to with similar emotion stances. Multiparty reciprocal laughter bouts provided affordances to build group coalition and strengthen the group's shared experiential stance.

In Ex. 4, during a handicraft activity, the teacher and children are modeling dough to make the three billy-goat shaped figures. The teacher is in a close proximity to the children; she instructs how to do the task and distributes a piece of dough to each child. One of the children, Joel, (on the teacher's left side) hits his dough, while laughing with excitement.

Ex. 4. Teacher; boys Joel, (5.2 y.); John (4.8 y.), Lucas (4.5 y.); a girl Agnes (4.8 y.).


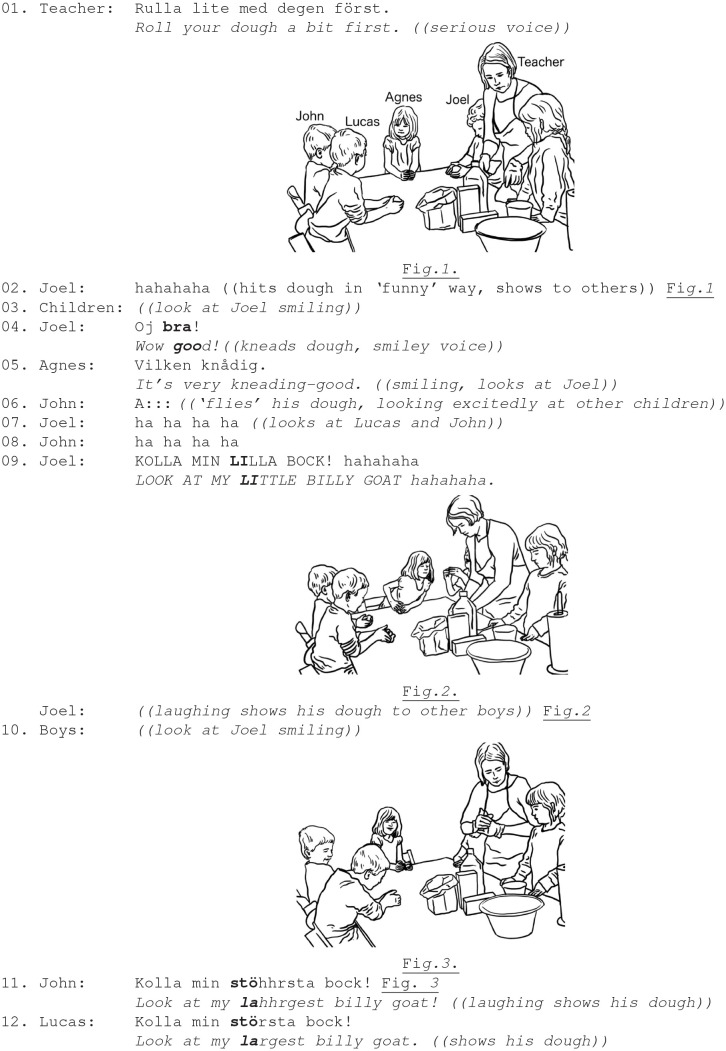


The children respond to the teacher's matter-of-fact instructions by rolling, playing and laughing about the billy-goat shapes of dough. Joel laughs while hitting his piece of dough (Fig. 1). The peer group are attentive to Joel's entertaining act: they look up, smiling, and join in his play actions (lines 3, 5, 6). When John playfully transforms his dough into a flying object, his entertaining act is appreciated: Joel's laughter invites affiliation and emotion sharing across the peer group (lines 6–12). The boys' laughing bout continues when Joel draws the other's attention to a new playful act with appended laughter tokens “*look at my little billy goat”* (line 9). By continuing play and laughter, the peer group members sustain their emotion sharing, strengthening and consolidating the in-group solidarity. John's talk is interspaced with laugher tokens, while he displays his piece of dough to the boys, using repetitive transformation “*look at my largest billy goat”* (line 11, Fig. 3). The teacher in this case continues her institutional task rather than paying attention to or disciplining the children's multiparty laughter and play.

### Children's Peer Laughter and Adult Disciplining: Resistance by Laughing and Joking

The children directed laughter toward peers and adults in situations when they committed some mild normative transgressions, e.g., painted wrongly, commented on food, or laughed at some aspects of the others' behavior. The children knew about rules and adults' insistence on obeying them, therefore they could find breaking the rules entertaining. In such cases, children's laughter strengthened the enjoyable potentials of the incongruent act that constituted a normative transgression from the institutional practice. When directed at the member of the peer group, children's laughter was reciprocated and involved group emotion-sharing and group coalition. If deemed as disruptive of the institutional activity, such peer-group laughter was not only ignored or rejected by the educators, but, together with normatively transgressive actions, it was evaluated by the teachers as situationally inappropriate and disruptive of the preschool activity. Notably, the teachers' management of children's emotional expressions invoked and brought forth the usually unspoken norms for normatively expected, attentive, and leveled actions.

In Ex. 5a–b, four 3–4 year-old girls are painting with water colors. Olivia and Lilly are splashing water around them, covering their hands in color and destroying the paper. The teacher repeatedly disciplines them mildly, but the girls do not comply.

Ex. 5a. Participants: Teacher; girls, Olivia (4,5 y.), Lilly (4,1 y.).


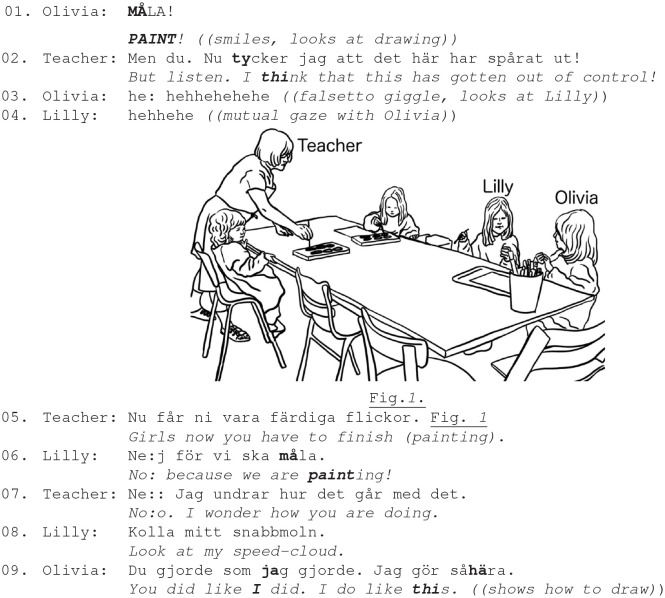


The teacher's disciplining comment “*I think that this has gotten out of control!”* is responded to with girls' exuberant reciprocal laughter, that expresses their shared emotional stance that strengthens their emotional group coalition and resistance toward the teacher's disciplining comment (lines 6–9, Fig. 1) (Bergson, 1911). The girls' peer laughter clearly displays their awareness and enjoyment of the situationally incongruent actions. It also achieves some teasing toward the teacher, who then uses a directive to close down the girls' activity *[“girls now you have to finish (painting)*”] but she only receives more resistance *(“no because we are painting”*) (lines 6; 8–9). Notably, the girls repeatedly initiate and reciprocate each other's laughter in ways that mark their enjoyment of inappropriate acts and playful resistance toward the teacher's attempts to control their actions.

Despite the teacher's disciplining, the girls continue their mischievous way of painting and use a lot of water. Olivia with very loud falsetto laughter and with a great deal of excitement displays her hands covered with color and instructs her friend how to do this clearly institutionally inappropriate kind of painting (line 1, Ex. 5b).

**Ex. 5b**


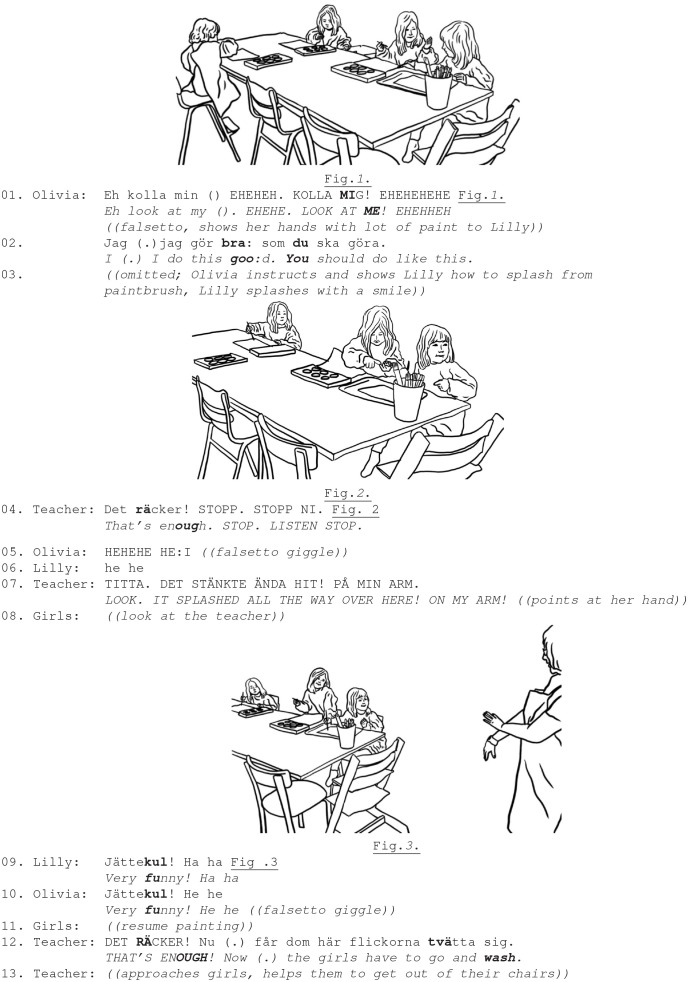


Both girls start splashing water with great excitement but the teacher disciplines them very loudly, demanding that they stop (line 4). They turn to the teacher and attentively observe her, but yet again, they respond to the teacher's reprimand by shared laughter (loud falsetto) that this time is directed at the teacher (lines 5–6, Fig. 2). This is an example of how children's laughter directed to the teacher is rejected and disciplined. In response to the girls' teasing laughter, the teacher upgrades her reprimand, loudly demanding them to witness the serious consequences of their misconduct “*look it splashed all the way over here”* (line 7), but to no avail. Through embodied means, the girls consolidate their playful resistance that clearly demonstrates an antithetical emotional expression and contrasts with the teacher's serious stance. The teacher then clearly rejects the girls' laughter and disciplines the girls' actions by finishing their drawing activity (line 12).

As demonstrated, the girls resist the teacher's serious and disciplining mode by engaging—inviting and sharing—a resistant, recalcitrant emotional stance, embellished with shared laughter (and a collective assessment “*very funny”* (lines 9–10). The children's shared laughter is embodied: its loudness, the girls' mutual gaze, and its position as a response to the teacher's disciplining directives show that girls engage in emotion coalition, and use laughter to achieve group affiliation by repeatedly resisting the teacher. The girls' laughter accomplishes both affiliative (laughing together) and disaffiliative work (laughing at the teacher's disciplining) with possible consequences for the social relationships within the group.

## Concluding Discussion

The present study has examined quantitative and qualitative patterns of 3–5 year-old children's and adults' laughter in a regular Swedish preschool. In our multi-method examination, we explored young children's embodied, interactional and affective engagements with the world, constitutive of and constituting shared norms and common ground in children's peer, and intergenerational encounters. The study contributes to a rather underexplored research area, namely young children's spontaneous laughter, its social functions, and peer group and adults' responses to it. This multi-method study reveals that young children's emotion sharing through laughter was a matter of generational—children's peer group—socialization.

It was found that children's laughter tended to be directed to children and adults' laughter tended to be directed to adults, meaning that laughter at the preschool was mainly a matter of peer interaction. Eighty seven percent of children's laughter was directed to other children (see Fig. 1), and adults directed their laughter to other adults 2.7 times as often as to children, providing that other adults were around (see Fig. 2). In addition to this, it was also found that children and adults exhibited different patterns of laughter. Children primarily sought and received affiliation through laughter in the peer group, and the adults were often focused on the institutional and educational goals of the preschool, i.e., securing the smooth flow of preschool activities. Intergenerational reciprocal laughter was a rare occurrence. This is illustrated by that fact that out of all the cases where a child laughed, only 2% of these involved an adult laughing in response.

These findings should not be interpreted as implying that the interaction between adults and children at the preschool was not characterized by warmth and respectfulness and that children and adults in laughter situations did not engage in affiliation and emotion sharing. In the following, we will discuss the results in detail.

### Adult Responses to Children's Laughter

As demonstrated, adults responded to children's laughter with a smile or other types of affirmation, and sometimes with laughter. When a child's laughter was directed to an adult recipient, the most common way for adults to respond was affirmation through smiling (30%), laughter (27%), or other means (12 %, Fig. 4). Notably, whereas adults' smiles confirm the positive emotion expressed by the child, it also means that the adult is not fully joining in with actual shared laughter where both parties are laughing (Ex. 2), although smiling responses do not reject the child's positive affective stance. Notably, adults' smiles in response to children's laughter do not interrupt the ongoing verbal activity and allow the adult to simultaneously affiliate with the child, and sustain the progression of the institutional activities. In all, smiling and affirmation through other means can be seen as a way of affiliating with emotion display, but with lesser intensity.

Adult responses to children's laughter were far from always a matter of affirmation and affiliation. In one third of adult responses to children's laughter, the response was not affirmative: adults did not respond at all (21%), or even explicitly rejected or opposed the child's laughter (10%, Fig. 4). Adults rejected and disciplined children when their laughter and actions were disturbing the institutional arrangements, and the qualitative analysis showed that such laughter could serve as a social resource for children's in-group solidarity, rapport, and shared sense of entertainment with peers (e.g., Bergson, 1911) (Ex. 5a; b). It was also found that children laughed more in situations where adults were not present, which implies that the presence of adults (who were usually organizing educational activities) has a constraining effect on children's tendency to laugh. Overall, the qualitative analysis showed that adults were concerned with preserving the smooth progression of institutional activities and modified their responses to children's laughter to fit these situational requirements, at times, modulating and regulating children's emotional expressions during their extended laughter bouts. In addition, the children's laughables were usually anchored in their peer group concerns, and could exhibit less potential for entertainment for adults.

The above findings, however, do not suggest that adults did not take part in socializing the children into positive emotion-sharing. Sometimes, the adults acted in ways that draw the children's attention to something entertaining and noteworthy, inviting their affective response (Ex. 2), but they did not reciprocate the children's laugher by laughing themselves. Such cases suggest that adults invited, and to some extent, provided guidelines for situationally appropriate displays of emotional stances. In this way, the adults also acted as socializing agents that instilled in children normative expectations and shared ways of demonstrating and reciprocating (or not) positive emotions. They took part in emotion socialization by providing institutionally approved interactional spaces for children's emotional displays of laughter. While the adults partially aligned with children's activities and emotional worlds, and displayed their understanding of what constitutes fun for the children, they also monitored the quality, duration, loudness, and content of the children's laughter and disciplined cases which they deemed to be inappropriate (Ex. 5a; b).

### Children's Peer-Directed Laughter

As demonstrated, in the preschool setting, the children direct more laughter to their peers than to adults (0.90 and 0.13 occurrences per minute, respectively, Fig. 3) and they laugh more when adults are absent compared to when they are co-present (0.90 and 0.54 occurrences per minute, respectively, Fig. 3). The qualitative analysis of the children's peer laughter showed that incongruency was a recurrent cause of laughter. The peer group members both provided a target of laughter, and were active recipients of laughter and emotion-sharing. Finding, identifying and picking up something for the other children to notice and emotionally share was done in an interactionally competent way even by young children. The object of laughter was clearly linked to the children's own activities (play, jokes, norm-breaking) and laughter was directed to the peers (Ex. 3; 4; 5a-b). The peers built up multiparty emotional affiliation; children's shared laughter could arise in situations where it became a way to establish and confirm a joint stance that was resistant toward the adult authority. Such laughables and playful acts attracted the peers' attention, and reciprocal laughter, smiling, repetition of playful acts, contributed to achievement of in-group solidarity (Bergson, 1911), common ground and peer group values.

As demonstrated, the children's laughter usually extended beyond a single instance. The multimodal interaction analysis revealed that even young children skillfully achieved a collective stance of rapport and funniness, as they initiated and shared it through publicly observable reciprocal laughter. Episodes of laughter did not follow a pre-determined trajectory, but were organized in an emergent way, by mild or louder, individual or collective, dispersed or coordinated laughter. The embodied features of the children's shared laughter show how laughter in the peer group was used in the pursuit and establishment of affiliation and rapport. Cascades of publicly and visibly shared laughter between the peers created an environment where the children organized their peer relationships (Goodwin, 1990), thereby constituting a significant emotion socialization power in a preschool context.

### Methodological Discussion

The present study has combined descriptive quantitative results and used them as a point of departure for detailed examination of the social characteristics and functions of children's and adults' laughter. Quantitative results provided an overall image of the recurrent patterns of how children and adults used laughter in preschool activities. The qualitative analysis allowed insights into the social organization, functions, and emotion-sharing potentials of laughter in and between the generations. The multimodal interaction analysis revealed how laughter served as a social resource for emotion-sharing and how it was an embodied matter (e.g., smiles and bodily orientation are easily missed in other types of data). Interaction analysis also allowed insights into the specific ways in which emotional expressions in social interaction were not simply an individual one-directional affair, rather they had a recipient from whom affiliation was sought. In all, through multimodal interaction analysis of participants' actions, it was possible to attend to psychological phenomena as shaped in human activity and intertwined with embodied social interaction (Goodwin, 2018).

Overall, the study suggests that laughter between generations is interesting in that it can be seen as indicative of how children and adults handle alterity (cf. Linell, 2009, p. 82) in their everyday life (a similar investigation in family settings can provide additional knowledge on emotion-sharing between children and parents). Laughter is thus not simply a matter of emotion affiliation and sharedness. By deploying multiple methods, the present study points to the importance of viewing emotion in social interaction not just as a matter of communicating an emotion from one person to another, but as an intricate process of inviting the others into or negotiating the common emotional and experiential ground.

## Ethics Statement

The study has been approved by Regional Ethics Board (Östergötland County). The data used for this study was collected as part of a larger research project subjected to ethical vetting by a regional committee for research ethics. Written and oral information was provided to staff and parents, and a consent form was signed for those adults who wished to participate (for parents, this consent also included their children). When visiting the pre-school, the researcher frequently asked the children's permission for recording, and the researcher was sensitive to signs of discomfort from the children that could be associated with being observed for the study. To avoid for the participants to be recognized, detailed information about the participants is not provided, and the sketches used for illustrative purposes are anonymized.

## Author Contributions

Both authors have contributed to the study, to qualitative and quantitative parts, and to the formulation and general description of the study.

### Conflict of Interest Statement

The authors declare that the research was conducted in the absence of any commercial or financial relationships that could be construed as a potential conflict of interest.

## Figures and Tables

**Table:** 

:	: prolonged syllable
AMP	:relatively high amplitude
(())	: further comments of the transcriber
?	: denotes rising terminal intonation
.	: indicates falling terminal intonation
**bro**	: sounds marked by emphatic stress are underlined
kommer	: indicates talk in Swedish
(.)	: micro pause
(0.5)	: pause length in seconds
*come*	: translation to English
[	: indicates overlap in talk or nonverbal acts

## Footnotes

^1^The study is also part of an ongoing research project that aims to further our understanding of the socialization of emotional expressions in Swedish everyday life settings, in preschools and homes. Financial grant from Swedish Research Council (Grant no: D0762601) is gratefully acknowledged (PI AC).

^2^Already at 10 months children very rarely laugh without a recognizable reason, (Kawakami et al., 2009).

^3^The project has been approved by Regionala etikprövningsnämnden i Linköping, Avdelning för prövning av övrig forskning, Linköping University, Hälsouniversitets kansli, Sandbäcksgatan 7, 581 83 Linköping, Sweden.

^4^A related finding from an unpublished quantitative analysis of the same data is that adult laughter in response to children's communicative actions that are designed to invite laughter is rare in the data overall: only 3% of all the instances of laughter.

^5^The illustrations (line drawings) are original and have not been published before. They are unique and are made specifically for the present research study.
